# Age-dependent parathormone levels and different CKD-MBD treatment practices of dialysis patients in Hungary - results from a nationwide clinical audit

**DOI:** 10.1186/1471-2369-14-155

**Published:** 2013-07-18

**Authors:** István Kiss, Zoltán Kiss, Csaba Ambrus, András Szabó, János Szegedi, József Balla, Erzsébet Ladányi, Botond Csiky, Ottó Árkossy, Marietta Török, Sándor Túri, Imre Kulcsár

**Affiliations:** 1 2nd Department of Internal Medicine Department Section of Geriatrics, Semmelweis University Faculty of Medicine, Budapest, Hungary; 2 Department of Nephrology-Hypertension, St Imre Teaching Hospital and B.Braun Avitum Hungary CPLC 1st Dialysis Centre, South-Buda Nephrology Centre, Halmi Street 20-22., Budapest H-1115, Hungary; 3 School for Ph.D. Candidates of Aesculap Academy, Budapest, Hungary; 4 2nd Department of Pediatrics, Semmelweis University Faculty of Medicine, Budapest, Hungary; 5 1st Department of Internal Medicine, Szabolcs-Szatmár-Bereg County Health Care Service Holding Corporation and 2nd Dialysis Centre of B. Braun Avitum Hungary CPLC, Nyíregyháza, Hungary; 6 1st Department of Internal Medicine, Debrecen University Medical School, Debrecen, Hungary; 7 Fresenius Medical Care Hungary Ltd., Budapest, Hungary; 8 Diaverum Hungary Ltd., Budapest, Hungary; 9 Department of Pediatrics, Szent-Györgyi Albert University Medical School, Szeged, Hungary; 10 4th Department of Internal Medicine, Markusovszky Hospital of Vas County and 6th Dialysis Centre of B. Braun Avitum Hungary CPLC, Szombathely, Hungary

**Keywords:** Calcium (Ca), Chronic kidney disease-mineral and bone disorder (CKD-MBD), Chronic kidney disease (CKD), End-stage renal disease (ESRD), Parathyroid hormone (PTH), Phosphate (PO_4_)

## Abstract

**Background:**

Achieving target levels of laboratory parameters of bone and mineral metabolism in chronic kidney disease (CKD) patients is important but also difficult in those living with end-stage kidney disease. This study aimed to determine if there are age-related differences in chronic kidney disease-mineral and bone disorder (CKD-MBD) characteristics, including treatment practice in Hungarian dialysis patients.

**Methods:**

Data were collected retrospectively from a large cohort of dialysis patients in Hungary. Patients on hemodialysis and peritoneal dialysis were also included. The enrolled patients were allocated into two groups based on their age (<65 years and ≥65 years). Characteristics of the age groups and differences in disease-related (epidemiology, laboratory, and treatment practice) parameters between the groups were analyzed.

**Results:**

A total of 5008 patients were included in the analysis and the mean age was 63.4±14.2 years. A total of 47.2% of patients were women, 32.8% had diabetes, and 11.4% were on peritoneal dialysis. Diabetes (37.9% vs 27.3%), bone disease (42.9% vs 34.1%), and soft tissue calcification (56.3% vs 44.7%) were more prevalent in the older group than the younger group (p<0.001 for all). We found an inverse relationship between age and parathyroid hormone (PTH) levels (p<0.001). Serum PTH levels were lower in patients with diabetes compared with those without diabetes below 80 years (p<0.001). Diabetes and age were independently associated with serum PTH levels (interaction: diabetes × age groups, p=0.138). Older patients were more likely than younger patients to achieve laboratory target ranges for each parameter (Ca: 66.9% vs 62.1%, p<0.001; PO_4_: 52.6% vs 49.2%, p<0.05; and PTH: 50.6% vs 46.6%, p<0.01), and for combined parameters (19.8% vs 15.8%, p<0.001). Older patients were less likely to receive related medication than younger patients (66.9% vs 79.7%, p<0.001).

**Conclusions:**

The achievement of laboratory target ranges for bone and mineral metabolism and clinical practice in CKD depends on the age of the patients. A greater proportion of older patients met target criteria and received less medication compared with younger patients.

## Background

The prevalence of chronic kidney disease (CKD), and consequently, dialysis therapy and appearance of co-morbidities, is continuously increasing in the developed world because of the high prevalence of well known risk factors and aging of the population. One of the most frequent complications of CKD is chronic kidney disease-mineral and bone disorder (CKD-MBD) [1,2]. In CKD-MBD, characteristic laboratory parameters (parathyroid hormone [PTH], calcium [Ca], and phosphate [PO_4_]) are strongly associated with mortality and morbidity. In clinical practice, there is a considerable amount of patients with laboratory parameters concurrently outside the normal target ranges, which further aggravates the risk of morbidity and mortality [3-6]. The appearance of cardiovascular diseases may also be caused by vascular calcification, which is strongly linked with disruptions in Ca and PO_4_ metabolism [7]. The lack of related evidence-based data also contributes to continuous changes in clinical guidelines [8-14].

One of the consequences of an aging population is that the vast majority of dialyzed CKD patients are older than 65 years old. As a result, renal replacement therapy has become part of geriatric medicine [15]. In elderly CKD patients, there are some special considerations, such as cognitive and functional impairment, falls, and bone fractures. Consequently, these conditions also require different treatment practice, and elderly CKD patients have different characteristics compared with younger age groups.

To determine CKD-MBD disease characteristics and treatment practice in Hungary and compare them with international data, we performed a survey among adult Hungarian dialysis patients to audit therapeutical practice. Our hypothesis is that there are different disease characteristics and treatment practices between patient groups according to age. We expect local health caregivers to treat their patients more individually and in general, to focus more on CKD-MBD issues, which has been observed internationally. According to a recent report, a focused 18-month campaign resulted in an increased proportion of patients who achieved serum Ca and PO_4_ targets by 24% and 8%, respectively [16]. In addition, the results of this survey should be helpful for improving patient education, the importance of which is also endorsed by international experience [17,18].

The first aim of this survey was to describe characteristics of patients regarding their CKD-MBD status in Hungary. Furthermore, we analyzed the characteristics of patients in different age groups. The second aim was to analyze the differences between laboratory test results of bone metabolism markers (PTH, Ca, and PO_4_) and prescribed therapies in different age groups. Finally, we compared our results with international experience and data, setting the necessary direction for development in Hungary.

## Methods

This was a multicenter observational survey. We enrolled 5335 chronic dialysis patients (for at least 3 months on renal replacement therapy) from all dialysis units in Hungary. (participating investigators and units are listed in the Acknowledgements.) Patients on hemodialysis (HD) and peritoneal dialysis (PD) were included. Patients with one or more basic values missing were excluded from the survey. The final dataset included 5008 patients. To identify age-dependent conclusions, we divided the study population into groups based on their ages (group I: <65 years; group II: ≥65 years).

The survey was performed between December 2010 and March 2011. We collected cross-sectional clinical, laboratory, and treatment data from the second quarter of 2010. Patient identity codes, sex, birth year, height and weight, cause of CKD, and the presence of complications, such as diabetes, bone disease, soft tissue calcification, and parathyroidectomy, were included in the dataset. Dialysis physicians completed an electronic survey for each patient regarding the presence of bone abnormalities. Bone abnormalities were defined as any radiologically documented bone fracture or other bone abnormality and vascular calcification or tissue calcinosis detected by any imaging technique. Laboratory parameters measured were serum intact parathyroid hormone (se-iPTH), serum PO_4_, Ca, and albumin levels. Samples were analyzed in different laboratories based on the location and dialysis provider's preference. In case of PTH, the vast majority of blood samples were analyzed by either the Elecsys (Roche Diagnostics, Meylan, France) intact PTH assay or the Architect (Abbott Diagnostics, Abbott Park, Il, USA) intact PTH assay. We also collected data on prescribed medications, including phosphate binders, vitamin D analogs, and calcimimetic agents. All laboratory- and treatment-related data were captured from the databases of dialysis providers.

Results are presented as mean ± standard deviation (SD) or in absolute and relative frequencies (%) for continuous and categorical variables, as appropriate. The levels of se-iPTH followed log-normal distribution, and therefore, we chose to use the geometric mean and 95% confidence interval (CI), as well as median and 1–3 quartiles. Differences between patient groups were compared using the Student's t-test or ANOVA. Tukey’s test was used for post-hoc analysis. The Kruskal–Wallis and Mann–Whitney U tests were used for continuous variables and the z-test for categorical variables. To demonstrate the association of se-iPTH with age, diabetes, and other parameters of dialysis patients, we analyzed the data by two-way ANOVA, multivariate linear regression, and multivariate logistic regression models. Statistical analysis was performed by the Statistica software program (version 10.0, Tulsa, Oklahoma, USA).

All patients were informed about details of the survey and gave written consent for the collection and scientific analysis of their individual data. This study was approved by the nationwide ethical authority (Medical Research Council Scientific and Ethical Committee [ETT-TUKEB]; approval number: 384/PI/2010; 5372-0/2010-1018EKU).

## Results

The mean age of the 5008 dialyzed CKD patients was 63.4±14.2 years and 47.2% were women. The patients were distributed almost equally between the two age groups (group I: n=2413 and group II: n=2595) (Table 1). The proportion of female patients was higher in the older group than in the younger group (52.8% vs 41.2%, p<0.001). Interestingly, mean body mass index was comparable in both age groups and sexes. The leading causes of ESRD were hypertensive nephrosclerosis (22.9%) and diabetic nephropathy (22.1%). Hypertensive nephrosclerosis, diabetic nephropathy, and tubulointerstitial disease were more prevalent in the older group than in the younger group (p<0.001), whereas the proportions of glomerulonephritis and polycystic disease were higher in the younger group than in the older group (p<0.001). Diabetes as a co-morbid condition was also more frequent in the older group than in the younger group (37.9% in group II and 27.3% in group I, p<0.001). A total of 38.6% of all patients had previously defined bone disease(s) and the proportion of soft tissue calcification was as high as 50.7%. Bone disease and soft tissue calcification were significantly more frequent findings in the older group than in the younger group (bone disease: 42.9% vs 34.1%, p<0.001; calcification: 56.3% vs 44.7%, p<0.001).

**Table 1:** Demographic and laboratory data of the two age groups

		**Total (n=5008)**	**Age-groups (years)**		** Comparison of groups**
			**I. <65 (n=2413)**	**II. ≥65 (n=2595)**	
Age (year)		63.4 ± 14.2	51.5 ± 10.1	74.5±6.3	-
Gender (%)	Male	2644 (52.8%)	1418 (58.8%)	1226 (47.2%)	*p*<0.001
	Female	2364 (47.2%)	995 (41.2%)	1369 (52.8%)	
BMI (kg/m2)	Male	26.8 ± 5,2	27.0 ± 5.7	26.7 ± 4.6	NS
	Female	26.4 ± 6,1	26.4 ± 7.0	26.4 ± 5.4	NS
Dialysis modality (%)	PD	569 (11.4% )	341 (14.1%)	228 (8.9%)	*p*<0.001
	HD	4439 (88.6%)	2072 (85.9%)	2367 (91.2%)	
intact PTH (pg/ml) (median, quartiles)		178.0 75.8–361.5	206.0 82.2–451.0	157.0 70.0–30.2	*p*<0.001
intact PTH (pg/ml) (geometric mean, 95% CI of mean)		158.7 153.5–169.1	184.9 175.7–194.6	136.7 131.6–143.8	*p*<0.001
Calcium (mmol/l)		2.25 ± 0.19	2.24 ± 0.21	2.26 ± 0.17	*p*<0.001
Phosphate (mmol/l)		1.50 ± 0.49	1.63 ± 0.50	1.39 ± 0.43	*p*<0.001
CaxP (mmol^2^/l^2^)		3.38 ± 1.10	3.63 ± 1.15	3.14 ± 0.99	*p*<0.001
Albumin (g/l)		39.4 ± 4.2	39.9 ± 4,3	38.9 ± 4.1	*p*<0.001
Diabetes mellitus (%)		1641 (32.8%)	658 (27.3%)	983 (37.9%)	*p*<0.001
Bone disease (%)		1935 (38.6%)	822 (34.1%)	1113 (42.9%)	*p*<0.001
Soft tissue calcification (%)		2540 (50.7%)	1078 (44.7%)	1462 (56.3%)	*p*<0.001
Parathyroidectomy (%)		104 (2.1%)	84 (3.5%)	20 (0.8%)	*p*<0.001
Cause of ESRD	Glomerulonephritis	813 (16.2%)	524 (21.7%)	289 (11.1%)	*p*<0.001
	Tubulointerstitial	695 (13.9%)	293 (12.1%)	402 (15.5%)	*p*<0.001
	Diabetes mellitus	1105 (22.1%)	455 (18.9%)	650 (25.1%)	*p*<0.001
	Hypertension	1146 (22.9%)	464 (19.2%)	682 (26.3%)	*p*<0.001
	Polycystic	379 (7.6%)	238 (9.9%)	141 (5.4%)	*p*<0.001
	Unknown	189 (3.8%)	99 (4.1%)	90 (3.5%)	NS
	other	681 (13.6%)	340 (14.1%)	341 (13.1%)	NS
Prescribed therapy (%)	Calcimimetic	259 (5.2%)	196 (8.1%)	63 (2.4%)	*p*<0.001
	Ca based PO_4_-binder	2011 (40.2%)	1033 (42.8%)	978 (37.7%)	*p*<0.001
	Non-Ca based PO_4_-binder	947 (18.9%)	626 (25.9%)	321 (12.4%)	*p*<0.001
	Native vitamin D	166 (3.3%)	89 (3.7%)	77 (3.0%)	NS
	Active vitamin D	2020 (40.4%)	1037 (43%)	983 (37.9%)	*p*<0.001
	None	1349 (26.9%)	489 (20.3%)	860 (33.1%)	*p*<0.001

*NS* non-significant.

A total of 569 (11.4%) patients were on PD and 4439 (88.6%) were on HD. The proportion of PD patients was lower in the older group than in the younger group (8.9% vs 14.1%, p<0.001). There were no differences in epidemiological or laboratory parameters between these patient groups. The prevalence of diabetes was similar (32.7%) between PD and HD patients. However, bone disease (39.9% vs 29.2%) and soft tissue calcification (52.7% vs 35.5%) were more frequent in HD than in PD patients (p<0.001). Separate analyses were conducted for the two patient groups, which resulted in similar results (data not shown). Therefore, we only reported results for the whole patient population.

Laboratory test results are summarized in Table 1. Serum iPTH and PO_4_ levels were significantly lower (p<0,001) in the older group than in the younger group. Serum calcium and albumin levels were also significantly different between age groups. However, we do not believe that these differences are clinically relevant.

**Figure 1:**
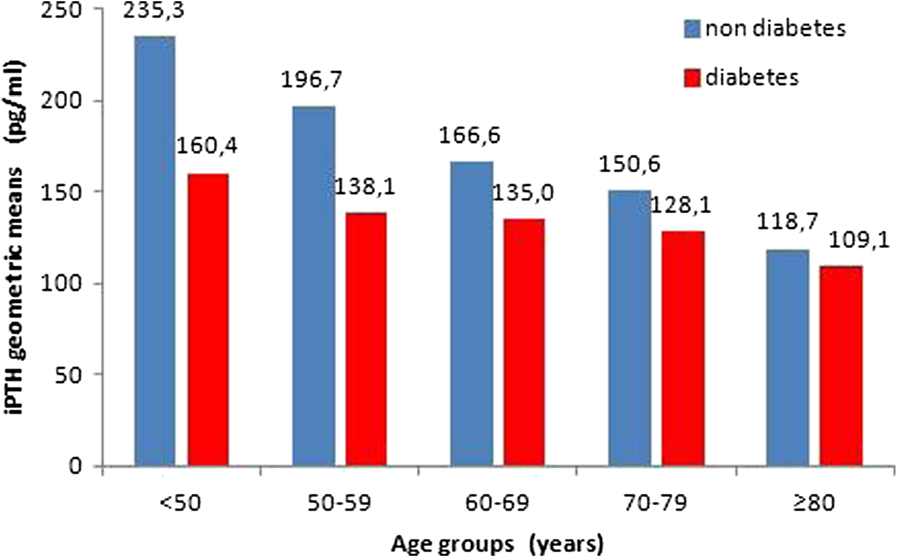
**Association between serum iPTH level, age, and diabetes. **Two-way ANOVA: DM p<0,001; age groups p<0,001; interaction DM x age groups p=0,138.

To further determine the associations among age, diabetes, and PTH levels, we divided the study population into five age groups (<50; 50–59; 60–69; 70–79; ≥80). Using these categories, we observed decreasing PTH levels with increasing age in both diabetes and non-diabetes subgroups (two-way ANOVA, p<0.001 for all comparisons; p=0.138 for the interaction diabetes × age) (Figure 1). Apart from the highest age group (≥80 years), se-iPTH levels were always significantly higher in the non-diabetes subgroup. In multivariate linear regression with PTH as the continuous dependent variable, age (b=-3.61, p<0.001), diabetes (b=-79.76, p<0.001), Ca (b=-145.13, p<0.001), and PO_4_ (b=165.76, p<0.001) were independent predictors of PTH. In logistic regression, age (10-year increments, odds ratio [OR] 1.07 [1.02–1.11], p<0.01), diabetes (OR 1.33 [1.17–1.51], p<0.001), serum PO_4_ levels (0.1 mmol/l increments, OR 0.92 [0.91–0.94], p<0.001) and serum Ca levels (0.1 mmol/l increments, OR 1.23 [1.19–1.27], p<0.001) were independent predictors of PTH levels <120 pg/ml (Table 2). In a similar model, age ≥65 years increased the risk of PTH <120 pg/ml by 33% (OR 1.33 [1.18-1.49], p<0.001).

**Table 2:** Predictors of iPTH<120 pg/ml using a multivariate logistic regression model

	**Odds ratio**	**95% CI**	**p**
Age (10 years increments)	1.07	1.02 – 1.11	<0.01
Ca (0.1mmol/l increments)	1.23	1.19 – 1.27	<0.001
PO_4_ (0.1 mmol/l increments)	0.92	0.91 – 0.94	<0.001
Gender (male vs female)	1.09	0.97 – 1.23	NS

*NS* non-significant.

The proportion of patients who achieved laboratory targets in the age groups is shown in Table 3. Although serum Ca levels were significantly higher in the older group (Table 1), more patients in this group reached the target range (p<0.001). Approximately half of the patients met the target criteria for PO_4_, and this was slightly higher in the older group than in the younger group (p<0.05). Similarly, a higher proportion of patients in the older group than in the younger group had their PTH levels in the reference range of 120–540 pg/ml (50.6% vs 46.6%, p<0.01). We also found a higher proportion of patients with PTH levels lower than 120 pg/ml in the older group compared with the younger group (40.2% vs 33.4%, p<0.001). If all three parameters were combined, the simultaneous achievement of targets in the younger group was 15.8%, which was significantly (p<0.001) lower than that in the older group (19.8%). The percentage of patients who achieved all three laboratory targets was only 17.9% (n=896). Serum Ca and iPTH levels in the patients are shown in Table 4. A total of 32.9% of patients achieved both laboratory targets.

**Table 3:** Numbers and proportions of patients who achieved laboratory targets in different age groups

**Laboratory parameters**	**<65 years (n=2413; 48.2%)**	**≥65 years (n=2595; 51.8%)**	**Comparisons of groups**
Ca (2.1-2.4)	1499 (62.1%)	1736 (66.9%)	p<0.001
PO_4_ (1.2-1.8)	1186 (49.2%)	1364 (52.6%)	p<0.05
iPTH (120–540)	1124 (46.6%)	1312 (50.6%)	p<0.01
Ca/PO_4_/PTH in the target range	382 (15.8%)	514 (19.8%)	p<0.001

**Table 4:** Number and proportion of patients based on their serum iPTH and Ca levels

		**Se–Ca (mmol/l)**			**Total**
		**<2.1**	**2.1-2.4**	**>2.4**	
Se– iPTH (pg/ml)	<120	208 (4.2%)	1172 (23.4%)	469 (9.4%)	1849 (36.9%)
	120-540	494 (9.9%)	1647 (32.9%)	295 (5.9%)	2436 (48.6%)
	>540	170 (3.4%)	416 (8.3%)	137 (2.7%)	723 (14.4%)
Total		872 (17.4%)	3235 (64.6%)	901 (18.0%)	5008 (100.0%)

The proportions of patients receiving different CKD-MBD-related medications are shown in Table 1. There was a considerable number of patients (n=1349; 26.9%) who were not prescribed any of these drugs. Older patients were less likely than younger patients (p<0.001) to receive any of the listed medications, with the exception of native vitamin D (cholecalciferol), which was prescribed to a minority of patients in both groups (3.7% and 3.0%). The majority of patients received Ca-based phosphate binders rather than non-Ca-based phosphate binders (40.2% vs 18.9%). The prescription of non-Ca-based phosphate binders was higher than Ca-based phosphate binders only in the subgroup with PTH levels >540 pg/ml and Ca levels >2.4 mmol/l. A total of 43.1% of patients with PTH levels <120 pg/ml and Ca levels >2.4 mmol/l were prescribed Ca-based phosphate binders. Among patients receiving phosphate binders, older patients were more likely to receive Ca-based binders (75.3%) compared with younger patients (62.3%). However, soft tissue calcification was more prevalent in the older group than in the younger group. While approximately 10% of patients in the high PTH group did not have therapy, only approximately 60% of those received active vitamin D analogs, and only 24.1% of hypercalcemic patients in this subgroup were treated with a calcimimetic agent. A total of 31.6% of hypercalcemic patients with PTH levels below the target range received active vitamin D analogs and some patients also received a calcimimetic agent in this PTH subgroup.

Treatment patterns are shown for the two age groups in Table 5. We found a higher prevalence of older patients than younger patients meeting CKD-MBD target values (19.8% vs 15.8%, p<0.001), despite a lesser need of therapy in this patient group. Considering patients who were within the target ranges for all parameters (Ca, PO_4_, and PTH), 30.7% did not require treatment in the older group, and this proportion was higher than that in the younger group (18.8%, p<0.001). When comparing patients who received treatment versus those who did not receive treatment in the older group, the proportion of patients meeting target criteria was not different for PTH (53.3% vs 50.4%, p: NS) and PO_4_ (52.7% vs 52.4%, p: NS). Patients who received treatment were slightly less likely to fall in the target range for Ca (64.9% vs 69.0%, p<0.05).

**Table 5:** Numbers of treated patients and prevalence of drugs prescription based on serum iPTH (pg/ml) and Ca (mmol/l) levels

**Therapy**	**iPTH<120 n=1849**		**iPTH 120–540 n=2436**		**iPTH>540 n=723**	
	**Ca≤2.4 n=1380**	**Ca>2.4 n=469**	**Ca≤2.4 n=2141**	**Ca>2.4 n=295**	**Ca≤2.4 n=586**	**Ca>2.4 n=137**
Calcimimetic	13 (0.9 %)	2 (0.4 %)	64 (3.0 %)	14 (4.7 %)	133 (22.7 %)	33 (24.1 %)
Ca-PO_4_ binder	567 (41.1 %)	202 (43.1 %)	886 (41.4 %)	95 (32.2 %)	223 (38.1 %)	38 (27.7 %)
Non Ca-PO_4_ binder	155 (11.2 %)	64 (13.6 %)	383 (17.9 %)	85 (28.8 %)	207 (35.3 %)	53 (38.7 %)
Native vitamin D	28 (1.5 %)	7 (0.4 %)	93 (3.8 %)	2 (0.08 %)	32 (4.4 %)	4 (0.6 %)
Active vitamin D analogs	324 (24 %)	143 (31.6 %)	964 (45.6 %)	155 (52.94 %)	359 (62.3 %)	75 (57.1 %)
No therapy	544 (39.4 %)	152 (32.4 %)	527 (24.6 %)	60 (20.3 %)	51 (8.7 %)	15 (10.9 %)

## Discussion

In this multicenter cross-sectional study among 5008 dialysis patients in Hungary, we found an inverse relationship between se-iPTH and age, and this was independent from the association between diabetes and iPTH. We also found that a higher proportion of older patients achieved treatment targets, whereas a lower proportion of these patients received therapy than in younger patients. In addition, our study showed that medication prescription practice did not follow current therapeutic guidelines in a significant proportion of patients in 2010.

More than half of our study population was aged older than 65 years. We showed that this significant proportion of patients was better controlled according to the CKD-MBD guidelines compared with younger patients, despite the lesser need for medication. Serum PO_4_ levels were lower in elderly patients than in younger patients, despite a lower use of Ca and non-calcium-based PO4 binders. This finding could reflect lower PO_4_ intake in elderly patients compared with younger patients. However, lower phosphorous could be a result of lower PTH levels in older patients.

Serum iPTH levels were lower in patients older than 65 years of age than in those younger than 65 years. Elderly patients were more likely to have PTH levels in the target range, and the prevalence of PTH levels below the target range was also higher in elderly patients. This finding is unlikely to be a result of overtreatment of secondary hyperparathyroidism because active vitamin D analogs and calcimimetic were less prevalent in the older group than in the younger group. Lower PTH levels in elderly patients could reflect a higher inflammatory state in these patients or the former prescription of aluminum-based phosphate binders. These findings are consistent with those in a previous study by Pelletier et al. [19] in a large cohort of HD patients in France.

An inverse association between age and PTH has been proposed in previous studies [20,21]. Iatrogenic factors may contribute to hypoparathyroidism. Elderly patients are more likely to be treated with Ca-based phosphate binders compared with younger patients. A relatively higher Ca burden may suppress PTH production. In addition, elderly patients are more likely to receive aluminum-based phosphate binders that also may aggravate hypoparathyroidism. Lower serum albumin concentrations in the older group suggests malnutrition and chronic inflammation, which is also associated with hypoparathyroidism. Insulin deficiency in type 1 diabetes might play a role in reduction of serum PTH levels. Furthermore, previous studies have shown that high glucose medium inhibits PTH secretion in cultured parathyroid cells [22,23]. Martinez et al. also reported a similar association between high glucose levels and hypoparathyroidism; however, others could not confirm this association [24]. Relative hypoparathyroidism is associated with adynamic bone disease, as well as with an increased risk of vascular calcification and bone fracture. Vascular calcification and bone fracture are known to dramatically increase mortality in dialysis patients. Therefore, we suggest that special attention should be paid to the management of elderly diabetic patients with hypoparathyroidism.

In our survey, elderly patients were more likely to meet treatment targets compared with younger patients on dialysis. Additionally, a much lower proportion of older patients than younger patients received medications. Protein intake, and consequently PO_4_ intake, are lower in older patients, leading to an increased prevalence of malnutrition. This could result in better adherence to a low PO_4_ diet, which is perceived as better phosphate control. A lower body mass of elderly patients may also result in more adequate dialysis. However, this factor might be of less importance in the current results. Because relative hypoparathyroidism is a more frequent finding in elderly patients than in younger patients, we consider that the higher prevalence of hyperparathyroidism in younger individuals could result in relatively better PTH control in the older population.

Based on our survey results, meeting se-iPTH targets was similar to that in the COSMOS study. [25]. A total of 14.4% of patients had hyperparathyroidism, which is a relatively low percentage. Despite the fact that approximately 80% of those patients did not have hypercalcemia, only half of those patients were taking active vitamin D analogs. A total of 36.9% of patients had se-iPTH levels below the target range, which is a great concern because those patients have a high risk for vascular and soft tissue calcification, as well as for bone fractures and mortality. If iPTH levels in our patients (300±386 pg/ml) are compared with those in a Japanese dialysis population (341±272 pg/ml) [26], we can conclude that in Hungary, patients are under tighter control; however, our standard deviation is higher. To decrease the proportion of hypoparathyroid patients, one potential treatment strategy could be the more frequent use of low calcium dialysate solutions, which is increasingly prevalent in Hungary.

With regard to target levels, it should be taken into consideration that current guidelines recommend a wider PTH target range than the previous guidelines, but some physicians may still manage patients according to the old guidelines and control patients more strictly, causing a higher prevalence of hypoparathyroidism. More than one third of our patients were below the lower limit of the PTH target range, which is not appropriate clinical practice. In general, among Hungarian dialysis patients, se-iPTH levels that are too low (<120 pg/ml) are far more frequent than (>540 pg/ml) se-iPTH levels that are too high. The most threatened patients (n=345; 6.9%) are those in whom se-iPTH and Ca levels are simultaneously out of target range. We conclude that a 20% yearly mortality rate [27] among dialysis patients might partly be caused by the high proportion of CKD-MBD patients who are frequently out of target laboratory ranges. Only 23% of patients with se-iPTH levels above the target range were on calcimimetic agents. Increasing the prescription of calcimimetics would be particularly important because of their high potential to decrease PTH levels without hypercalcemic side effects. Furthermore, calcimimetics also increase the sensitivity of the parathyroid gland to vitamin D. The combination of the two drugs is particularly beneficial for patients with increased serum PTH and Ca levels. Strict local financial regulations are likely to contribute to the low percentage of calcimimetic use. Non-Ca-based PO_4_ binders are far less frequently used than Ca-based PO_4_ binders. This treatment pattern is most likely influenced by the strict rules of drug coverage.

The majority (64.6%) of patients (n=3235) were within the normal Ca target range (2.1 -2.4 mmol/l) and there were only 901 patients (18%) who were hypercalcemic. With respect to international data, this result is comparable because in the COSMOS study [28], the percentages of patients in the target range were 55% and 28% [29] for serum Ca and PTH levels, respectively. We took the recent KDIGO guideline into account, which allows for a wider normal range of PTH levels. In Macedonia, 79% of patients were within the serum Ca target range. However, in this study, the target range was wider (2.1–2.6 mmol/l) than the target range that we used in the current study [30].

In our survey, the percentage of patients who were treated with native vitamin D was extremely low (3.3%). Considering the highly prevalent vitamin D insufficiency in the general population and also in CKD patients, all dialysis patients should be presumed as being vitamin D deficient. An increasing amount of evidence suggests supplementing dialysis patients with native vitamin D without even measuring their vitamin D status.

Our study has several limitations. A cross-sectional survey cannot examine changes in laboratory parameters in time because of changes in treatment strategy. Therefore, our findings regarding treatment practice should be evaluated carefully. Our survey was based on a single blood sample for each patient, not allowing for possible large intra-individual variation of laboratory parameters. Different laboratory assays were used to measure se-iPTH levels. However, we believe that the difference between the assays used did not greatly affect our results and conclusions. Our survey did not capture possible important confounding variables, such as dialysis vintage, co-morbidities, residual renal function, and dialysis adequacy, which also could have influenced the examined associations. Despite these limitations, this nationwide survey provides important information about our patient population and it is able to show directions to further improve the quality of dialysis care.

## Conclusions

In summary, this was the first survey in Hungary in which almost all patients on maintenance HD or PD were enrolled to analyze patient characteristics and treatment practices related to CKD-MBD. We found significant age-related associations in ESRD patients. We confirmed the inverse association between age and serum iPTH levels, and this was independent of the presence of diabetes. Oder patients were more likely to achieve laboratory targets than younger patients. Older patients were prescribed less medications related to CKD-MBD than younger patients and there was a high percentage of older subjects not receiving any related medication. The majority of ESRD patients had unbalanced Ca and PO_4_ metabolism. However, the target achievements of laboratory parameters are comparable with international data. We found a high prevalence of relative hypoparathyroidism in Hungary. Last, universal administration of native vitamin D, more frequent prescription of calcimimetics, and non-Ca-based PO_4_ binders based on current guidelines would be beneficial. Further longitudinal research needs to be performed to determine the effect on morbidity and mortality of age-related differences of CKD-MBD in dialysis patients.

## Abbreviations

CKD: Chronic kidney disease; CKD-MBD: Chronic kidney disease-mineral and bone disorder; ESRD: End-stage renal disease; DM: Diabetes mellitus; se-iPTH: Serum intact parathyroid hormone; Ca: Calcium; PO4: Phosphate; KDIGO: Kidney Disease Improving Global Outcomes.

## Competing interests

The authors declare that they have no competing interests.

## Authors’ contributions

All authors were involved in design, conception, and acquiring data of this study. IK, ZK, CA, and IK were involved in statistical analysis, writing, and editing the manuscript. All authors critically revised each version of the manuscript and then approved the final manuscript.

## Pre-publication history

The pre-publication history for this paper can be accessed here:

http://www.biomedcentral.com/1471-2369/14/155/prepub

## Members of the CKD-MBD Working Group of the Hungarian Society of Nephrology

Katalin Albert (Budapest, DVH), Csaba Ambrus (Budapest, BBAH), Ottó Árkossy (Budapest, FMC), József Balla (Debrecen, FMC), Attila Benke (Veszprém, BBAH), Klára Berta (Budapest, FMC), Béla Borbás (Debrecen, BBAH), Mária Bőke (Vác, FMC), Botond Csiky (Pécs, FMC), Ilona Egerváriné Mezei (Hatvan, FMC), Sándor Ferenczi (Győr, BBAH), Mária Haraszti Magdolna (Kecskemét, FMC), Judit Harsányi (Budapest, FMC), Mária Hensperger (Mátészalka, BBAH), Szilvia Kazup (Siófok, BBAH), Éva Kiss (Keszthely és Szeged, DVH), István Kiss (Budapest, BBAH), Zoltán Kiss (Budapest), Aranka Koós (Ózd, FMC), Dezider Kósa (Zalaegerszeg, DVH), László Középesy (Cegléd, FMC), Imre Kulcsár (Szombathely, BBAH), Erzsébet Ladányi (Miskolc, FMC), Katalin Magyar (Baja, DVH), Lajos Major (Esztergom, FMC), Miklós Mihalcsó (Szentes, FMC), Márta Molnár (Szigetvár, FMC), Attila Nagy (Sátoraljaújhely, FMC), Lajos Nagy (Tatabánya, BBAH), Péter Nagy (Eger, FMC), Anikó Németh (Nagykanizsa, FMC), József Németh (Dunaújváros, BBAH), Attila Orosz (Budapest, DVH), Nóra Pálvölgyi (Sopron, FMC), Éva Pató (Budapest), Kálmán Polner (Budapest), Sarolta Repetics (Budapest), Jenő Rédl (Szolnok, DVH), Csaba Rikker (Budapest, FMC), Antal Rozinka (Kisvárda, BBAH), Saeb Knita (Szikszó, FMC), András Szabó (Budapest), Tamás Szabó (Kistarcsa, BBAH), János Szegedi (Nyíregyháza, BBAH), Tamás Szelestei (Kaposvár, BBAH), Béla Tichy (Kiskunfélegyháza, FMC), Eszter Tóth (Békéscsaba, Gyula, BBAH), Marietta Török (Karcag, DVH), Sándor Túri (Szeged), Erzsébet Varga (Hódmezővásárhely és Kalocsa, DVH), Gábor Varga (Ajka, BBAH), Tünde Varga (Salgótarján, FMC), Gyula Wágner (Szekszárd, BBAH), Gábor Zakar (Székesfehérvár, BBAH), and György Zsigmond (Budapest). BBAH: B. Braun Avitum Hungary CPLC. DVH: Diaverum Hungary, Ltd. FMC: Fresenius Medical Care Dialyzis Center, Ltd.
